# ReSleeve or revisional one anastomosis gastric bypass for failed primary sleeve gastrectomy with dilated gastric tube: a retrospective study

**DOI:** 10.1007/s00464-023-10609-6

**Published:** 2023-12-06

**Authors:** Wadie Boshra Gerges, Ahmed S. M. Omar, Ahmed Ain Shoka, Mohammed Abdalmegeed Hamed, Hossam S. Abdelrahim, Fady Makram

**Affiliations:** Department of General Surgery, Faculty of Medicine, Ain Shams University, Cairo, Egypt

**Keywords:** LSG, Weight loss failure, Revisional bariatric surgery, Resleeve, Laparoscopic one anastomosis gastric bypass, Dilated gastric tube

## Abstract

**Background:**

Revisional bariatric surgery (RBS) has been increasingly performed due to weight loss failure (WLF). Many revisional procedures have been proposed after primary laparoscopic sleeve gastrectomy (pLSG) failure, including ReSleeve gastrectomy (ReLSG), and laparoscopic one anastomosis gastric bypass (LOAGB). Choosing the RBS post-pLSG failure represents a challenge. WLF without gastric tube (GT) dilation is undoubtedly converted to a malabsorptive procedure, but the presence of GT dilation makes it more difficult to select a RBS. This study aimed to compare two relatively simple revisional procedures after pLSG failure with dilated GT to help decision making on which procedure better done to which patient.

**Methods:**

Data of 52 patients who completed one year follow-up (FU) after their RBS (ReLSG: 27 or LOAGB: 25) for their failed pLSG were collected, assessed, correlated to weight loss (WL) and compared.

**Results:**

Mean operative time was 97 ± 18.4 min. with revisional LOAGB (RLOAGB) and 62 ± 11 min. with ReLSG. Six patients (11.5%) had seven postoperative procedure-specific complications. Significant hemorrhage occurred in three patients. Two cases of leakage were encountered with each procedure. LOAGB Patients had lower mean final weight (76.2 ± 10.5 vs 85.3 ± 13), lower mean Final BMI (26.4 ± 2.5 vs 29.7 ± 2.9) and higher mean percentage of excess weight loss (EWL%) (83.6 ± 13.5% vs 60.29 ± 14.6%). All RLOAGB patients and 77.8% of ReLSG patients had EWL% > 50%. RLOAGB patients had higher EWL% compared to ReLSG (*p* < 0.001). Insufficient WL (IWL) patients had higher EWL% compared to weight regain (WR) patients (*p* = 0.034).

**Conclusion:**

Both procedures (RLOAGB and ReLSG) were relatively safe and effective in terms of WL. RLOAGB led to higher WL compared to ReLSG in all types of patients despite higher Caloric intake. IWL patients had more WL compared to WR patients. WL was not related to GT dilation type. Large-scale longer-FU studies are still needed.

**Trial registration:**

PACTR202310644487566 (retrospectively registered).

Since its acceptance as a standalone primary bariatric procedure (BP), laparoscopic sleeve gastrectomy (LSG) progressively gained popularity until it became the most commonly worldwide performed BP [1]. Good short-term weight loss (WL) results of LSG, its relatively simple technique and reduced short and long-term complication rates contributed to it being preferred over more complex procedures [2, 3]. However, practice has shown that sleeve gastrectomy (SG) may fail and recent data on LSG long-term outcomes reported failure rates of up to 30% [4, 5]. Revisional bariatric surgery (RBS) has been increasingly performed due to weight loss failure (WLF), gastroesophageal reflux disease (GERD) and/or postoperative complications and overall revision rate was estimated to be 19.9% [6, 7].

Bariatric literature describes two types of WLF [7–12]: insufficient weight loss (IWL) and weight regain (WR) after initial successful WL. Although IWL and WR definitions of are not standardized, most studies defined IWL as achieving < 50% of excess weight loss (EWL) over a period of 24 months [7–12]. WR had different definitions including > 25% of EWL [8–12], increase of 10 kg or more from WL nadir [11, 12], and 15% total WR from nadir [8]. Literature also describes two types of gastric tube (GT) dilation after SG [7, 10, 13–16]: primary dilation (defined as a large upper gastric fundal pouch) and secondary dilation [defined as homogenous/uniform GT dilation with residual gastric volume (RGV) > 250 mL]. GT dilation may be attributed to technical errors during primary SG or to a natural process of GT dilation [7, 10, 13–16].

Many revisional procedures have been proposed after primary LSG (pLSG) failure, including ReSleeve (ReSG), Roux-en Y gastric bypass (RYGB), bilio-pancreatic diversion with duodenal switch (BPD-DS), and recently, One anastomosis gastric bypass (OAGB) [13, 17, 18]. The choice of the RBS following LSG failure represents a challenge; there is still no consensus on which revisional BP is better for which patients [13, 17, 18]. The discovery of a possible GT dilation or the persistence of fundus encouraged the practice of ReSG with the rationale of resizing the GT using surgical staplers when dilation is proven radiologically [9, 14–16, 19–21].

After restriction failure, adding malabsorption seems to be an accepted solution for further long-term WL [4, 13, 17, 22, 23]. OAGB is a technically less demanding malabsorptive BP that involves a single side-to-side anastomosis between a lesser curvature-based long-sleeved gastric pouch and the jejunum with biliopancreatic limb (BPL) ranging from 150 to 250 cm [1, 24]. OAGB has shown excellent long-term results (high rates of WL, comorbidity resolution and patient satisfaction) with low mid and long-term complications rates [24–32]. Many studies have reported OAGB as a potent well-tolerated revisional option for failed restrictive procedures [23, 25, 33–38].

WLF without GT dilation is undoubtedly converted to a malabsorptive BP, but the presence of GT dilation and the persistence of part of fundus with its secreted hormones makes it more difficult to select a RBS. This study aimed to compare two relatively simple revisional procedures after SG failure with dilated GT to help decision making on which procedure better done to which patient.

## Methods

### Study design

This retrospective comparative study included patients who had revisional laparoscopic OAGB (RLOAGB) or laparoscopic ReSleeve (ReLSG) after failed pLSG with dilated GT in the General Surgery Department (Bariatric Unit), Ain shams University Hospitals. The patients’ data of the RBS were collected prospectively from October 2019 to August 2023, and the data of pLSG were collected retrospectively by questionnaires on patients’ first presentation. Each patient was followed up for at least one year. An informed consent was taken from all patients including the surgical procedure, its possible complications and alternatives and the enrollment of their data in the study. The study was approved by the institutional research ethics committee (IRB No: 0006379).

### Indications for revisional surgery

RBS was performed to fit patients with WLF after at least 18 months from their pLSG. Two types of failure were considered: IWL [Percentage of EWL (EWL%) is < 50% within 2 years after LSG] and WR (regaining 25% of EWL from nadir after initial successful WL). Patients with severe gastroesophageal reflux disease (GERD) (severe symptoms, oesophageal erosions or Barrett) were offered RYGB instead.

### Eligibility criteria for the study

The study included patients who underwent RBS (RLOAGB: group-A or ReLSG: group-B) after failed LSG with radiological evidence of GT dilation, either diffuse (RGV > 250 mL) or fundus dilation (with or without GT diffuse dilation) and were followed up for minimum one year. Patients with pLSG complicated by leak and patients who had Laparoscopic RYGB (LRYGB) within the first year due to complications (refractory BR or severe stricture) were excluded.

### Preoperative assessment

**Fig. 1:**
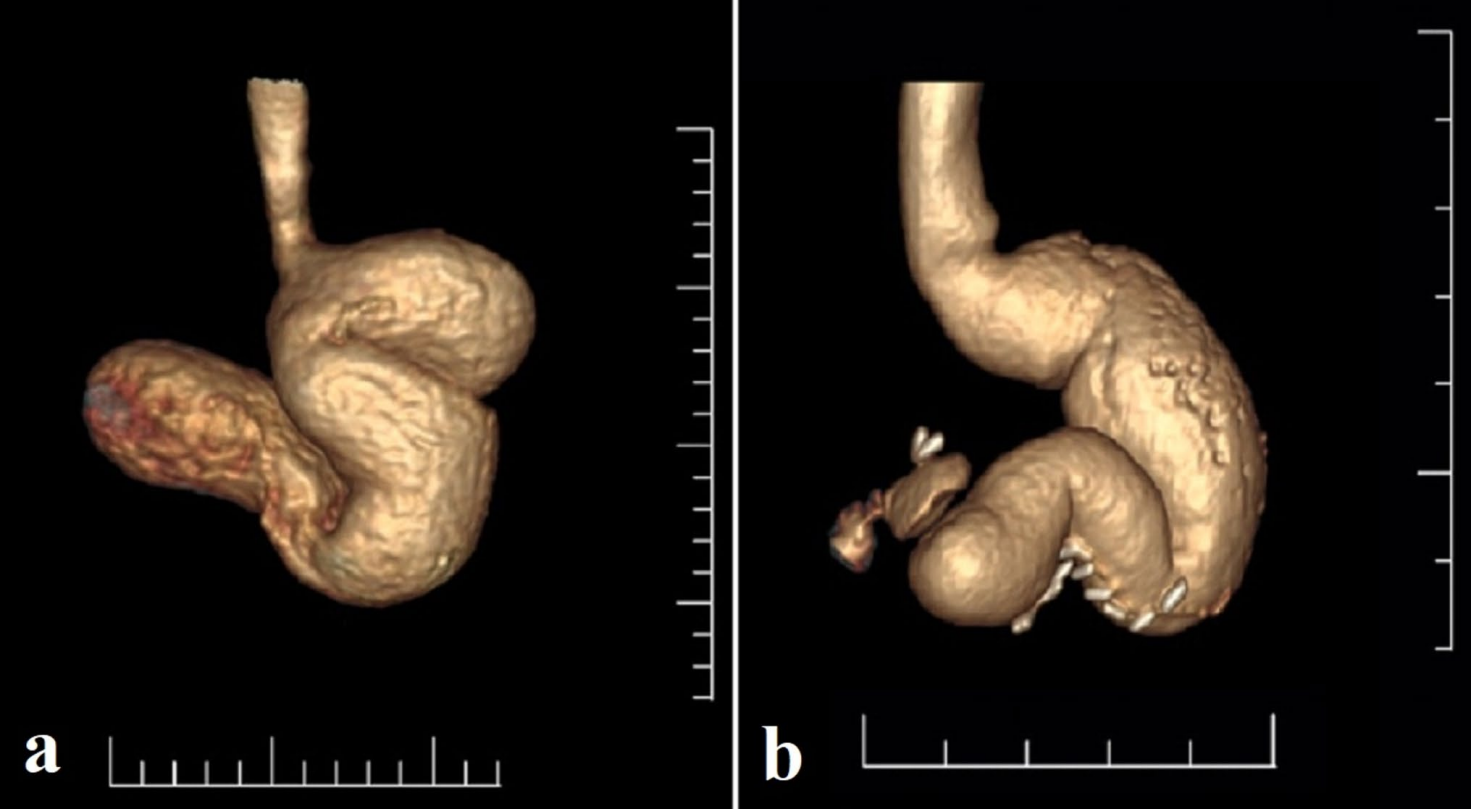
CT gastric volumetry of two cases showing: **a** GT with primary dilation, **b** GT with primary diffuse dilation

Detailed history was taken from all patients including dietary habits, medical comorbidities and previous treatments for morbid obesity (MO) including the pLSG. The data of pLSG were collected through questionnaires: primary preoperative weight (before pLSG), average daily caloric intake (ADCI) and least recorded (Nadir) weight after pLSG. Preoperative weight was measured and BMI calculated. Abdominal ultrasound was done to detect gall bladder stones if present. All patients had esophagogastroscopy to exclude oesophageal erosions or Barrett, gastritis and ulcers. GT dilation type (diffuse or fundal pouch) was assessed by computed tomography (CT) scan gastric volumetry; diffuse dilation was considered with RGV > 250 mL (Fig. 1).

### Surgical procedure

All procedures were performed in our bariatric surgery unit by its staff members with standardized techniques. Patients with concomitant gall bladder stones had cholecystectomy before revisional bariatric steps. Using the Harmonic™ scalpel (Ethicon), adhesions to the GT were lysed dissecting the posterior gastric fold, the residual fundus and the herniated fundus in the hiatal opening (if present). The wide hiatus (when detected) was repaired using 2/0 Ethibond Excel® (Ethicon).

**Fig. 2:**
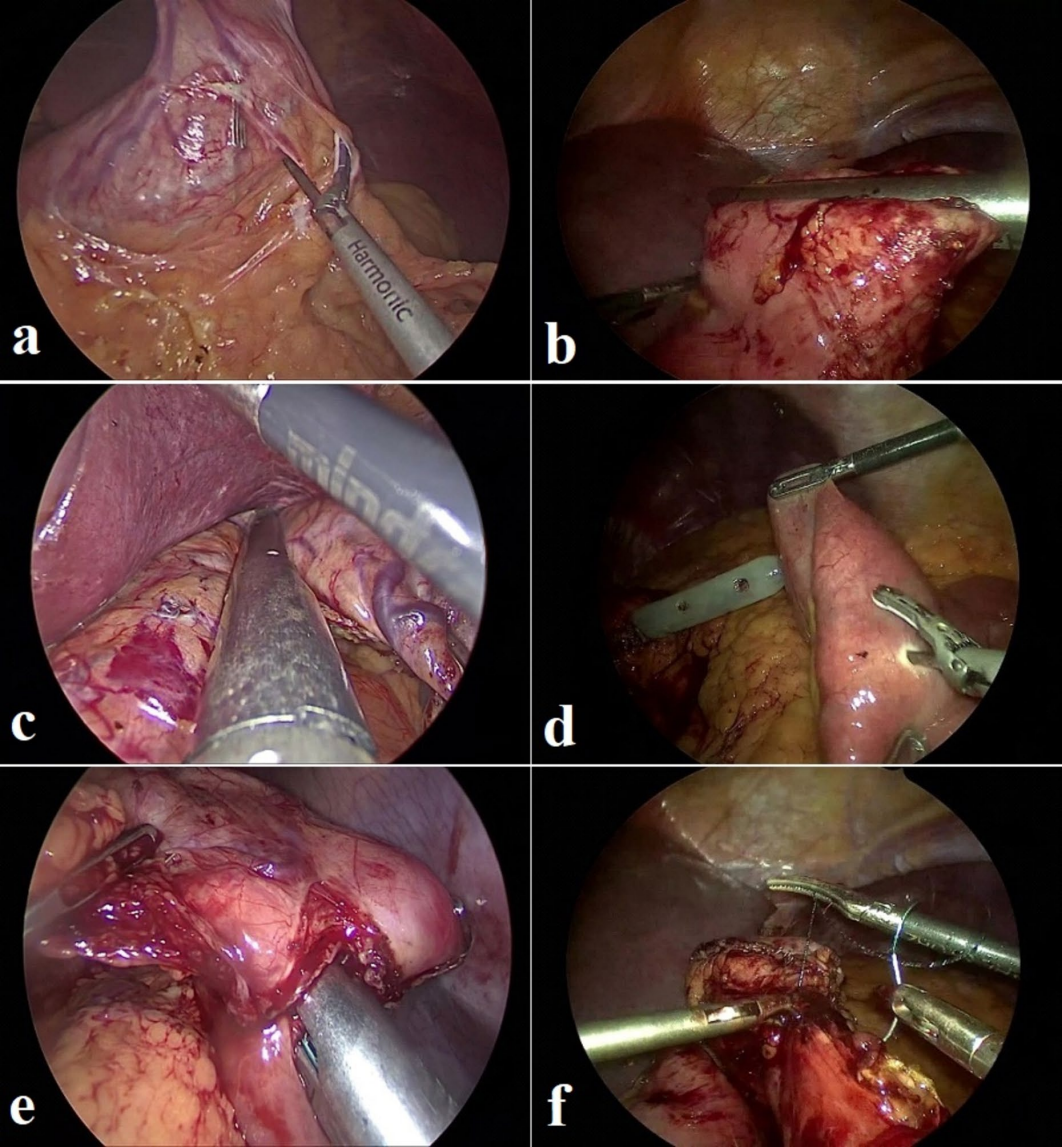
Steps of one case of RLOAGB: **a** adhesiolysis, **b** transverse division of gastric tube, **c** longitudinal resizing of gastric pouch, **d** enterotomy, **e** stapled side-to-side gastrojejunostomy, **f** closure of common opening by V-loc

In group-A: A window in the lesser omentum was created at the incisura level, followed by transverse division of the GT using Echelon Flex 60™ Staplers with black and green reloads (Ethicon) and resizing the gastric pouch on 36 Fr bougie to form a narrow longitudinal lesser curve based pouch reaching the incisura. An antecolic side-to-side gastrojejunostomy (GJ) was created between the gastric pouch and the jejunum 200 cm from the Treitz ligament using gold/green reloads. The common GJ opening was closed with 2/0V-Loc™ (Covidien) (Fig. 2).

**Fig. 3:**
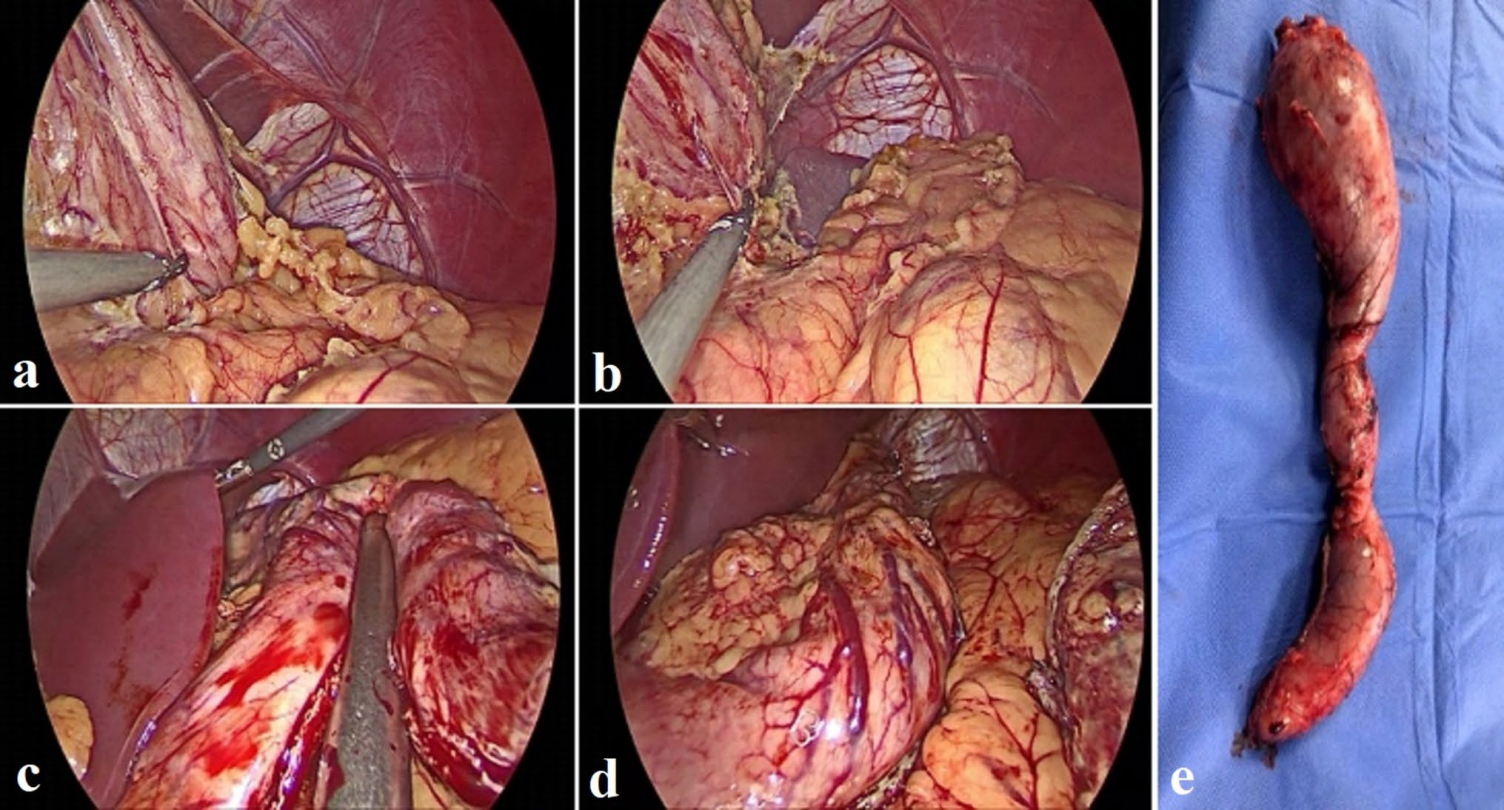
Steps of one case of ReLSG: **a** adhesiolysis of GT posterior wall, **b** adhesiolysis and dissection of fundus and hiatus, **c** longitudinal stapling of GT, **d** intraoperative methylene blue leak test, **e** the excised part of GT

In group-B: After complete adhesiolysis, a resleeve was done on a 36 Fr bougie using Echelon Flex 60™ Staplers with black and green reloads (Ethicon), starting from the pylorus 4 cm away from the sphincter upward to 1 cm away from the angle of His with removal of excised gastric tissue (Fig. 3).

### Postoperative management and follow up (FU)

Early ambulation was encouraged, and oral fluid intake was allowed on postoperative day (POD) 2 after excluding leakage with oral dye series. Patients were discharged home after confirming their well-being and tolerance to oral fluids. Patients were seen on weekly basis for one month to assess tolerance to oral intake and to detect possible early complications. CT of abdomen and pelvis with oral contrast was done when leakage was suspected. All patients were advised to take oral supplements containing iron, calcium, vitamins B12 and D together with oral proton pump inhibitor daily for the first 6 months. RLOAGB patients were prescribed life-long multivitamins. FU visits were scheduled at 3, 6 and 12 months postoperatively to assess WL.

### Data collection, management and statistical analysis

The following data were collected: patients’ sex, age at the time of the revisional procedure, medical comorbidities, height, initial preoperative weight and BMI (before pLSG), Nadir weight after LSG, EWL% of the pLSG, ADCI after pLSG using specially formulated questionnaires, second preoperative weight and BMI (before RBS), indication for surgery (IWL/WR), timing between LSG and revision, preoperative radiological GT anatomy (diffuse/fundus dilation), operative time (OT), hospital stay and complications (bleeding and leak). At the end of the study period, these data were collected (by physical attendance or telephone questionnaires): FU period of each patient, ADCI (excluding the first postoperative month) and final weight. BMI and EWL% at time of final data collection were calculated.

The collected data were revised, coded, tabulated and introduced to a PC using Statistical package for Social Science (IBM Corp. Released 2017. IBM SPSS Statistics for Windows, Version 25.0. Armonk, NY: IBM Corp). Shapiro wilk’s test was used to evaluate normal distribution of Quantitative variables. Student’s *t* test was used to compare quantitative variable between two study groups. Categorical variables were compared using the Chi-square or Fisher exact test. Pearson correlation was used to assess the strength of association between two quantitative variables. Multivariate linear regression (MLR) analysis was used to determine which variables were associated independently with outcome variable. A *p* value < 0.05 was considered statistically significant.

## Results

Fifty-two patients completed the one year FU period required to be included (25 in group-A: RLOAGB, 27 in group-B: ReLSG). The mean FU duration was 23.6 ± 6.8 (12–38) months in group-A and 23.8 ± 6.4 (13–35) months in group-B with no significant difference. Table 1 shows the demographic and preoperative patients’ characteristics of at time of revision; all parameters showed no statistical differences between both groups.

**Table 1:** Comparison between patients of both groups regarding the demographic and preoperative characteristics

	Group A: RLOAGB (25)		Group B: ReLSG (27)		*p*	Sig
	Mean ± SD	Range	Mean ± SD	Range		
Age at time of RBS	37.4 ± 8.96	24–55	33.5 ± 8.98	22–55	0.127*	NS
Preop wt 1 (before pLSG)	139.44 ± 22.75	102–187	137.81 ± 19.52	109–182	0.784*	NS
BMI of preop wt 1	48.24 ± 5.58	38.6–58.4	48.16 ± 4.79	40.3–60.3	0.955*	NS
Nadir wt (after pLSG) (Kg)	100.68 ± 19.13	67–138	99.73 ± 16.89	75–139	0.852*	NS
BMI Of nadir	34.87 ± 5.65	23.4–44.4	34.86 ± 5.04	27.2–47.9	0.997*	NS
EWL% 1 (after pLSG)	55 ± 21.71	21.8–108.5	55.11 ± 17.01	29.5–86.1	0.984*	NS
ADCI 1 (after pLSG) kcal	1766.4 ± 180.0	1450–2080	1790 ± 182.36	1470–2170	0.790*	NS
Preop wt 2 (before RBS) (kg)	113.28 ± 17.33	80–147	111 ± 15.38	89–149	0.621*	NS
BMI of preop wt 2	39.2 ± 4.12	31.7–46	38.8 ± 3.73	32.9–48.2	0.712*	NS
Timing bet two operations (mon.)	34.28 ± 10.28	18–57	32.23 ± 8.28	19–49	0.436*	NS
Sex						
Male	9 (36%)		9 (33.3%)		0.84^‡^	NS
Female	16 (64%)		18 (66.7%)			
Type of failure						
IWL	11 (44%)		14 (51.9%)		0.571^‡^	NS
WR	14 (56%)		13 (48.1%)			
Type of dilatation						
Diffuse	19 (76%)		17 (63.0%)		0.309^‡^	NS
Pouch	6 (24%)		10 (37.0%)			
Medical comorbidities	13 (52%)		11 (40.7%)		0.416^‡^	NS

*Student test

^‡^Chi-Square Tests

All procedures were completed laparoscopically. Hiatal repair was performed for the detected wide hiatus in three and five patients in groups A and B, respectively. Mean OT (after excluding timing of cholecystectomy when performed) was 97 ± 18.4 min. (55–175) in group-A and 62 ± 11 min (30–95) in group-B. Most patients were discharged on POD 3; mean hospital stay was 3.7 (2–9) days.

Six patients (11.5%) suffered from seven major postoperative complications. Significant postoperative hemorrhage that necessitated blood transfusion occurred in three patients. In group-A, two patients (8%) had anastomotic leakage. One patient was presented with abdominal pain and fever and was diagnosed at POD 10 to have leakage in a subphrenic abscess that was drained percutaneously. The other patient presented acutely at POD 7 with pain and vital instability and CT confirmed leakage and peritonitis, and was urgently explored laparoscopically with peritoneal lavage, omental patching and feeding jejunostomy. Further supportive management helped to close the fistulae in both patients within 4–6 weeks. In group-B, also two patients (7.4%) had staple line leakage that was diagnosed on POD 5 and 7. Both patients were successfully managed with fully covered metallic stents and percutaneous drainage without surgical intervention.

During FU, 16 patients (64%) noticed non troublesome diarrhea/steatorrhea after RLOAGB. In group-A, 11 patients had preoperative GERD: 2 patients noticed increased/new symptoms, 5 patients noticed improved/disappeared symptoms. In group-B, 14 patients had preoperative GERD: 6 patients noticed increased/new symptoms. No patients had improved symptoms.

There was no significant difference between groups A and B as regard all postoperative parameters (Table 2) except for final weight and BMI, and final EWL%. Group A patients had lower mean final weight (76.2 ± 10.5 vs 85.3 ± 13), lower mean Final BMI (26.4 ± 2.5 vs 29.7 ± 2.9) and higher mean EWL% (83.6 ± 13.5% vs 60.29 ± 14.6%). All group-A patients had EWL% more than 50%, while only 21 patients in group-B (77.8%) had similar results. In the last 2 years of the study period, some early-operated patients (one in group-A and four in group-B) noticed some WR.

**Table 2:** Comparison between patients of both groups regarding the postoperative results

	Group A: RLOAGB (25)		Group B: ReLSG (27)		*p*	Sig
	Mean ± SD	Range	Mean ± SD	Range		
Mean FU duration (mon)	23.62 ± 6.84	12–38	23.81 ± 6.42	13–35	0.945*	NS
ADCI 2 (after RBS) kcal	1747.6 ± 175.7	1420–2170	1684.6 ± 168.39	1390–2060	0.197*	NS
Final wt (kg)	76.2 ± 10.5	59–107	85.3 ± 13	62–112	0.008*	**HS**
BMI of final wt	26.4 ± 2.5	22.4–33.4	29.7 ± 2.9	24.2–35	0.001*	**HS**
EWL%	83.7 ± 13.5	51.1–110	60.3 ± 14.6	24.2–92.5	0.001*	**HS**
Operative complications						
Leak	2 (8%)		2 (7.4%)		1.0^‡‡^	NS
Bleed	1 (4%)		2 (7.4%)		1.0^‡‡^	NS
GERD worsen or develop	2 (8%)		6 (22.2%)		0.252^‡‡^	NS
GERD Improve or disappear	5 (20%)		0		0.02^‡‡^	S

*Student test

^‡‡^Fisher exact test

Bold value represents significant results

Different personal and clinical parameters that might affect WL were statistically correlated to EWL% (Table 3). There was no significant association or correlation between final EWL% and all these factors and variables. Using multiple linear regression, after adjustment of relevant variables (Table 4), it was shown that type of RBS, EWL% after pLSG, ADCI before RBS, type of failure, and ADCI after RBS were the independent factors significantly related to EWL% after RBS. Group A patients had higher EWL% compared to group-B (regression coefficient = 28.8, *p* < 0.001, CI 21.94–35.82). IWL patients had higher EWL% compared to WR patients (regression coefficient = 16.38, *p* = 0.034, CI 1.33–31.43). EWL% was not related to type of GT dilation. Caloric intake after RBS was significantly related to EWL% between both groups, i.e., group-A patients had significantly higher ADCI despite higher EWL%.

**Table 3:** Relation between EWL% and patients’ personal and clinical parameters

Categorical variables	EWL%		p*	Sig
	Mean	± SD		
Sex				
Male	69.79	21.55	0.578	NS
Female	72.82	16.49		
Comorbidity				
No	72.60	21.32	0.730	NS
Yes	70.80	14.51		
Type of failure				
IWL	70.22	21.47	0.564	NS
WR	73.22	14.87		
Type of dilatation				
Diffuse	71.12	18.97	0.721	NS
Pouch	73.13	17.17		
Complications				
No	71.78	18.61	0.978	NS
Yes	71.57	17.35		

Continuous variables	*r* of EWL%	p**	Sig
Age	0.060	0.675	NS
Preop wt 1	− 0.092	0.519	NS
BMI of wt 1	− 0.102	0.477	NS
Nadir wt	− 0.153	0.283	NS
BMI of nadir	− 0.165	0.247	NS
EWL% 1	0.173	0.226	NS
ADCI 1	0.149	0.297	NS
Preop wt 2	− 0.137	0.337	NS
BMI of wt 2	− 0.174	0.221	NS
Timing bet two operations	− 0.007	0.959	NS
ADCI 2	0.023	0.872	NS

*Student test

**Pearson correlation

**Table 4:** Multivariate linear regression to study independent factors affecting EWL% after RBS

	Regression coefficient	*p*	Sig	95.0% Confidence interval for regression coefficient	
				Lower bound	Upper bound
RLOAGB^a^	7.525	0.080	NS	− 0.942	15.993
Group	**28.881**	**0.0001**	**HS**	21.941	35.822
Preop wt 1	− 0.300	0.327	NS	− 0.912	0.311
EWL% 1	**0.659**	**0.006**	**HS**	0.200	1.118
ADCI 1	**0.049**	**0.007**	**HS**	0.014	0.084
Preop wt 2	0.380	0.364	NS	− 0.456	1.215
IWL^b^	**16.378**	**0.034**	**S**	1.329	31.428
Type of dilatation(diffuse)^c^	0.636	0.881	NS	− 7.912	9.183
ADCI 2	** − 0.054**	**0.002**	**HS**	− 0.087	− 0.021
FU duration (months)	− 0.420	0.120	NS	− 0.954	0.114

^a^ReLSG is reference

^b^WR is reference

^c^Pouch is reference

Bold value represents significant results

Each group patients were divided into four subgroups according to the types of failure and GT dilation, and the EWL% in each subgroup was calculated and compared between both study groups (Table 5). EWL% was higher in group-A [significantly in all subgroups but insignificantly in the subgroup (WR with Pouch)].

**Table 5:** Comparison between RLOAGB and ReLSG groups among the four subgroups of patients as regard EWL%

	Group A: RLOAGB		Group B: ReLSG		*p*	Sig
	No	Mean EWL% ± SD	No	Mean EWL% ± SD		
IWL with pouch	4	90.80 ± 8.93	7	63.60 ± 17.87	0.02*	S
IWL with diffuse	7	87.21 ± 9.08	7	48.10 ± 12.70	0.0001*	HS
WR with pouch	2	77.80 ± 6.65	3	68.67 ± 10.80	0.357*	NS
WR with diffuse	12	80.21 ± 16.77	10	64.40 ± 9.78	0.021*	S

*Student *t* test

## Discussion

LSG failure generally falls into two categories [5, 12]: WLF (either IWL or WR) and GERD. Recent long-term studies of pLSG show higher than expected failure and revisional surgery rates with incidence of WR ranging from 14 to 37%. The pooled revision rates due to WLF and GERD were estimated as 13.1% and 2.9%, respectively [4, 6, 7, 39]. This study focused on two types of revisional procedures post-LSG failure in terms of WL and did not include post-LSG complications.

Multiple explanations have been speculated regarding the GT dilation. Most primary dilation cases are probably due to technical failure during pLSG with incomplete resection of the gastric fundus [12–16, 19–21, 40]. Many studies reported that incomplete fundus removal could be the actual cause of the detected dilated fundus and described that complete dissection of the fundus posterior aspect may be technically demanding and almost impossible in some extremely obese patients [20, 40]. In some cases of incomplete fundus removal, a small HH may not be identified during the pLSG, in which some gastric folds may be missed [16, 19, 20, 41]. Secondary GT dilation is usually due to false calibration with a large bougie during pLSG or due to the physiologic GT dilation [12–16, 19–21, 40]. Based on radiological studies using CT volumetry, a RGV threshold of 250 cm^3^ has been proposed as a possible indication for ReLSG below which the conversion to a malabsorptive BP is encouraged [16, 42, 43].

Revisional surgery is often burdened by higher rates of complications [13, 17, 44] and no standardized guidelines have been developed in literature for choosing a RBS after SG failure [13, 15, 17, 45, 46]. In patients with severe GERD symptoms (the main cause for revision), literature confirmed that RYGB should be the ideal option [4, 13, 17, 46]. In compliance with this, we excluded severe GERD patients from having these procedures.

ReLSG has been proposed as a feasible RBS after pLSG failure when a residual fundus is evident or when the GT shape suggests an improper technique [13–21]. Some short-term papers reported good WL results comparable to RYGB [41, 47]. Ambiguous data have been reported about ReLSG complications [15]; some series [16, 47] reported high GL rates, while others [21, 41] did not report any. ReLSG offers several advantages (compared with malabsorptive procedures) that encourage its practice: less technically challenging nature of procedure, increased restriction, decreased acid production, maintaining GI continuity, avoiding dumping and decreased risks of anemia, osteoporosis, protein and vitamin deficiency [15–17, 20, 21, 40, 48]. The negative effects of ReLSG include the increased risk of gastric leak (GL), the high-pressure system leading to onset/aggravation of GERD. Other disadvantages include the absence of malabsorptive effect and the resleeved GT being prone to re-enlargement with time causing insufficient WL with higher probability of long-term WR [4, 13, 15, 17, 40, 41, 46].

After failure of the restrictive SG, adding malabsorption has been proven an effective means for further WL [4, 13, 17, 22, 23, 46]. OAGB has been introduced and established as a viable alternative to the classic RYGB due to its relative technical simplicity, shorter learning curve and the ease of reversibility [1, 24–32]. Long-term studies demonstrated OAGB as an efficient primary BP that provides durable WL with acceptable complication rates [24–28]. Studies comparing primary OAGB to RYGB revealed some advantages with OAGB, such as shorter OT, fewer major complications (leakage and IH) and equal or even higher efficacy in WL [29–32]. Furthermore, OAGB, specifically with a 200-cm BPL, is believed to cause marked fat and sweets intolerance and is more malabsorptive than standard RYGB owing to its longer BPL, without reaching the malabsorptive dangers of BPD/DS [23, 35–38, 48–51].

Recently published systematic reviews and meta-analyses showed that RLOAGB is a valuable choice after failed restriction and that SG conversion to OAGB was technically easier [37, 49–54]; this would be particularly useful in handling revisional surgery and could be helpful in higher BMI patients. These studies demonstrated better WL and acceptable incidences of the main complications with RLOAGB compared to those of RYGB.

The current concerns existing for OAGB are the risks of postoperative malnutrition and bile reflux (BR) [25, 29, 36, 53, 55]; both are still debated [53–55]. Symptomatic BR, requiring revision, has been reported [23, 27, 36, 38]. Felsenreich et al. [50] study revealed better outcomes for OAGB than for RYGB in terms of acid exposure, even though more OAGB patients suffered from GERD symptoms; this may be a hint for the symptoms not being acid-based, but related to BR. The YOMEGA study [29] reported more reflux in the gastric pouch in RLOAGB compared to RYGB without difference in quality of life (QOL). While De Luca et al. [25] showed that the rates of symptomatic BR were lower than first feared. Tolone et al. [56] study showed significant anti-reflux effects of OAGB compared to SG. A comparative study [31] did not show procedure-specific advantages in GERD remission.

Two recent meta-analyses [53, 54] showed that OAGB has different effects on GERD where GERD resolution after converting restrictive surgery to OAGB was described by some studies, while others described de-novo emergence of GERD and BR in patients with no preoperative GERD symptoms. However, the incidence of severe BR requiring conversion to RYGB was low. In addition, most symptomatic patients experienced marked improvements by medications [53, 54].

In our study, three patients in each group (12% with RLOAGB and 11.1% with ReLSG) encountered seven postoperative intra-abdominal complications. Significant hemorrhage occurred in three patients (two with ReLSG and one with RLOAGB). With RLOAGB, two patients (8%) had anastomotic leakage. With ReLSG, also two patients (7.4%) had GL. Leakage cases were managed successfully with adequate drainage and optimization of general conditions. Our complications rate was considered relatively high when compared to other similar studies; this may be explained by the wide variability in experience of the operating surgeons. AlSabah et al. [41] and Omarov et al. [21] studies showed no early postoperative complications with ReLSG while Antonopulos et al. [47] series showed GL of 8.2%. With RLOAGB, Chiappetta et al. [37] and Jamal et al. [34] studies did not observe any leak or bleeding. Poublon et al. [36] series had 1.1% early intra-abdominal complications Alsabah et al. [57] reported three (10.3%) morbidities (two leaks and one stenosis). In Rheinwalt et al. [51] study, leak rate was 4.9%.

With our ReLSG after 23.8 months of FU, mean weight and BMI decreased from 111 ± 15.4 kg and 38.8 ± 3.7 to 85.3 ± 13 kg and 29.7 ± 2.9, respectively, and mean EWL% was 60.3 ± 14.6%. Only 21 patients (77.8%) had EWL% > 50%. Noel et al. [14] reported on 36 ReLSG patients with mean EWL% of 58.7% at 19.9-months FU. Rebibo et al. [15] series showed mean EWL% of 71.3% after 1 year. Silecchia et al. [16] achieved EWL% of 53.4% after 24 months. Nedelcu et al. [19] study patients’ mean BMI decreased from 38.1 to 29.8 after mean FU of 20 months. In Antonopulos et al. [47] study, mean BMI decreased from 40.5 to 31.6, mean EWL% was 69.5%.

With our RLOAGB after 23.6 months of FU, mean weight and BMI decreased from 113.3 ± 17.3 kg and 39.2 ± 4.1 to 76.2 ± 10.5 kg and 26.4 ± 2.5, respectively, and mean EWL% was 83.6 ± 13.5%. All patients had EWL% > 50%. Our greater efficacy might be related to the 200 cm BPL and pouch resizing done in all patients. At 24 months FU in Debs et al. [55] study of RLOAGB, mean weight and BMI decreased from108.83 kg and 40.1 to 77.8 kg and 28.9, respectively, and mean EWL% was 84.1%. Mean EWL% at 1-year FU was 64% in Chiappetta et al. [37] study, 60% in Poghosyan et al. [23] study and 58.9% in Alsabah et al. [57] study. In Jamal et al. [34] study, 58% of patients achieved EWL% of > 50% at 19-months FU.

Our study focused on WL outcomes, thus it excluded the cases that needed early revision due to complications (before one year) to LRYGB such as ReLSG complicated by non-dilatable stricture and RLOAGB complicated by severe BR. Minimal FU period was one year and mean FU period was nearly 2 years; this did not allow accurate study of WR after revision. After ReLSG, six cases noticed increased/newly developed GERD symptoms, no cases had improved symptoms. In literature, many ReLSG series showed aggravated/de-novo GERD symptoms [15, 17, 40, 41].

After RLOAGB, two cases (8%) noticed increased/newly developed GERD symptoms, while five cases noticed improvement/disappearance of symptoms. Kermansaravi et al. [49] study showed three new-onset GERD symptoms (13%). In Debs et al. [55] study, seven pts (9%) developed de-novo GERD. In Rheinwalt et al. [51] study, preoperative GERD was ameliorated in 86.7% of RLOAGB cases. New-onset reflux appeared in only one patient. Rayman et al. [38] reported GERD in 17.4% patients. Poghosyan et al. [23] reported the 8.3% de-novo GERD.

Patients who underwent RLOAGB reached lower weight and BMI than those who had ReLSG. Statistical analysis also showed that RLOAGB patients had significantly higher EWL% compared to ReLSG patients despite higher Caloric intake. IWL patients had higher EWL% compared to WR patients. EWL% was not related to the GT dilation type with non-significant trend of better response with diffuse GT dilation. Trying to define possible better indications for each procedure, our patients were divided into four subgroups according to the types of failure and GT dilatation. In all subgroups, RLOAGB resulted in significantly higher WL than ReLSG, except in (WR with fundal pouch) subgroup, the difference was insignificant; this type of patients can be offered both procedures. Larger-scale studies are required.

Studies comparing ReLSG with RLOAGB as revisional procedures and studies analyzing WL results in relation to types of WLF or preoperative GT anatomy are scarce. Rebibo et al. [15] showed that performing ReLSG for WR was associated with higher WL compared with IWL. In patients with RGV < 350 mL and with IWL, a malabsorptive procedure could possibly provide better results than those obtained with ReLSG. Al-Sabah et al. [41] showed that patients responded better if ReLSG was performed for IWL rather than WR. Sista et al. [58] study compared SG revision to OAGB vs RYGB and showed that WR patients responded better than IWL patients did in both types of GB. It also showed that OAGB gave better results, particularly in patients with IWL.

As RLOAGB was more effective than ReLSG in WL, ReLSG is better reserved for patients with GT volume of > 350 ml, as recommended by Rebibo et al. [15], who suggested that such a volume should be the new adopted cut-off, as it allowed more WL with less complications. There are many points regarding RLOAGB that require further research [53, 54]: the most suitable BPL length and associated nutritional deficiencies, the need for pouch resizing, and the relation to BR. If additional hiatoplasty during both procedures affects GERD symptoms or if the low-pressure system of OAGB is a good solution for GERD patients are not yet clarified [25, 37, 48–51, 56].

Our study Limitations include being retrospective, relatively short duration, and small patients’ number. The study did not include nutritional assessment (albumin, vitamins and trace elements), assessment of medical comorbidities resolution and QOL, and postoperative endoscopic surveillance. Crude and subjective assessment of GERD and ADCI was another limitation.

## Conclusion

Both procedures RLOAGB and ReLSG were relatively safe and effective in terms of WL. RLOAGB led to higher WL compared to ReLSG in all types of patients despite higher Caloric intake. IWL patients had more WL compared to WR patients. WL was not related to the type of GT dilation. Further large-scale longer-FU studies are still needed.
